# Right ventricular global dysfunction score: a new concept of right ventricular function assessment in patients with heart failure with reduced ejection fraction (HFrEF)

**DOI:** 10.3389/fcvm.2023.1194174

**Published:** 2023-08-04

**Authors:** Jan Benes, Martin Kotrc, Peter Wohlfahrt, Katerina Kroupova, Marek Tupy, Josef Kautzner, Vojtech Melenovsky

**Affiliations:** ^1^ Department of Cardiology, Institute for Clinical and Experimental Medicine-IKEM, Prague, Czech Republic; ^2^ Radiodiagnostic and Interventional Radiology Department, Institute for Clinical and Experimental Medicine-IKEM, Prague, Czech Republic

**Keywords:** right ventricular function assessment, right ventricular size, right ventricular dysfunction, heart failure, outcome

## Abstract

**Background:**

Right ventricular (RV) function is currently being evaluated solely according to the properties of RV myocardium. We have tested a concept that in patients with heart failure with reduced ejection fraction (HFrEF), RV assessment should integrate the information about both RV function as well as size.

**Methods:**

A total of 836 stable patients with HFrEF (LVEF 23.6 ± 5.8%, 82.8% males, 68% NYHA III/IV) underwent echocardiographic evaluation and were prospectively followed for a median of 3.07 (IQRs 1.11; 4.89) years for the occurrence of death, urgent heart transplantation or implantation of mechanical circulatory support.

**Results:**

RV size (measured as RV-basal diameter, RVD_1_) was significantly associated with an adverse outcome independent of RV dysfunction grade (*p* = 0.0002). The prognostic power of RVD_1_ was further improved by indexing to body surface area (RVD_1_i, *p* < 0.05 compared to non-indexed value). A novel parameter named RV global dysfunction score (RVGDs) was calculated as a product of RVD_1_i and the degree of RV dysfunction (1–4 for preserved RV function, mild, moderate and severe dysfunction, respectively). RVGDs showed a superior prognostic role compared to RV dysfunction grade alone (ΔAUC >0.03, *p* < 0.0001). In every subgroup of RVGDs (<20, 20–40, 40–60, >60), patients with milder degree of RV dysfunction but more dilated RV had similar outcome as those with more severe degree of RV dysfunction but smaller RV size (all *p* > 0.50), independent of tricuspid regurgitation severity and degree of pulmonary hypertension.

**Conclusion:**

RV dilatation is a manifestation of RV dysfunction. The evaluation of RV performance should integrate the information about both RV size and function.

## Introduction

Echocardiographic evaluation of right ventricular (RV) function is complicated due to its complex geometry. Nevertheless, a correct RV function assessment is crucial as RV dysfunction is associated with an adverse outcome in multiple pathologic conditions including pulmonary artery hypertension and heart failure (1–4). RV function plays an especially important role in patients undergoing LV-mechanical circulatory support implantation (5).

Traditional parameters for RV function assessment (TAPSE, Sm-TDI, fractional area change—FAC) are currently being replaced by more sophisticated measures (RV strain) (6, 7). However, all these parameters focus solely on the properties of RV myocardium, but RV size as well may have prognostic value in HFrEF patients (8). As both the size and the degree of RV dysfunction are related to the prognosis of HF patients, we propose that RV dilatation should be viewed as a manifestation of RV dysfunction.

The goal of the study was to test a novel concept that the information about RV size and function should be integrated into one parameter that would offer more accurate information about RV disease.

## Methods

### Study subjects

This is a retrospective analysis of prospectively enrolled patients; subjects with stable HFrEF (LVEF <40%) of at least 6 months duration (i.e., signs or symptoms of HF and LVEF <40% at least 6 months before enrollment and ongoing signs/symptoms of HF and ongoing LVEF < 40% at the time of enrollment) were enrolled in the study between 2008 and 2016 and prospectively followed. In all subjects, LVEF was assessed by echocardiography. Patients had to be on stable medical therapy for at least three months. Those with potentially reversible LV dysfunction (planned valve surgery, revascularization, or tachycardia-induced cardiomyopathy) were excluded. Patients were followed until July 2019.

Echocardiography and blood sample testing were performed upon enrollment. The protocol was approved by the Institutional Ethics Committee, and all subjects signed an informed consent. Patients were prospectively followed and the adverse outcome was defined as the combined endpoint of death, urgent heart transplantation, or ventricular assist device implantation. Due to the fact that time to non-urgent transplantation reflects donor availability rather than recipient's condition, patients who received a non-urgent heart transplant were censored as having no outcome event at the day of transplantation, as previously reported (9).

### Echocardiography

**Figure 1:**
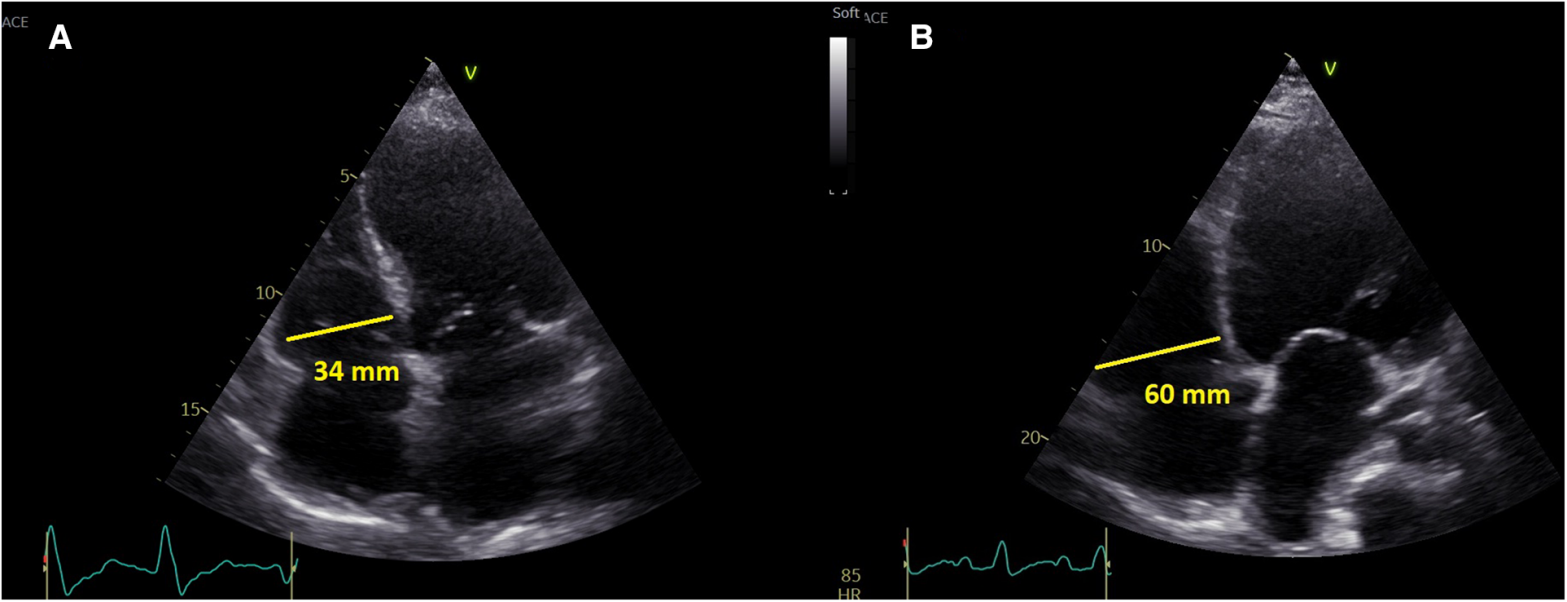
A representative presentation of non-dilated right ventricle (left) and dilated right ventricle (right).

Left ventricular size was measured in parasternal long axis (PLAX) as end-diastolic diameter, LV ejection fraction was assessed by the Simpson method (10). Right ventricular size was measured in apical 4-chamber view (A4C) as RV-basal diameter (RVD_1_) (11). All sonographers were (as per institutional protocol) instructed to obtain the A4C projection with interventricular and interatrial septum perpendicular to the probe and to obtain the image of the “heart cross” with best available quality (Figure 1). RV dilatation was formally defined as RVD_1_ > 42 mm, but in the analysis it was used as a continuous variable.

Right ventricular dysfunction was quantified semiquantitatively (preserved RV function, mild, moderate and severe RV dysfunction). The assessment of RV function was performed in an apical 4-chamber view by using tricuspid annular systolic excursion (M-mode TAPSE) (12) and tissue systolic velocity (Sm) (13) with the following cutoffs: normal RV function: TAPSE >20 mm, Sm >12 cm/s; mild RV dysfunction: TAPSE 16–20 mm, Sm 9–12 cm/s; moderate RV dysfunction: TAPSE 10–15 mm, Sm 6–9 cm/s; severe RV dysfunction: TAPSE <10 mm, Sm <6 cm/s. In case of disagreement between TAPSE and Sm, qualitative visual estimation of RV motion in apical 4-chamber was also taken into account. Similarly, in patients with the history of pericardial opening and decreased parameters reflecting longitudinal RV function, RV radial contraction was incorporated into the RV function assessment as well. Mitral and tricuspid regurgitation severity was assessed semi-quantitatively in three degrees (mild-moderate-significant) (14). Vivid-7 and Vivid-9 (General Electric, Milwaukee, Wisconsin) were used for echocardiographic study. Indexation of RVD_1_ was performed using body surface area (BSA) that was calculated as BSA = 0.007184. weight (kg)^(0.425). height(m)^(0.725).

### Nuclear scintigraphy

RV end-systolic and end-diastolic volumes (and subsequently RV ejection fraction) was assessed using electrocardiogram-gated three-dimensional equilibrium Tc-labeled blood pool single photon emission computed tomography (SPECT). Patients received an injection of stannous pyrophosphate (Technescan PYP, Curium, the Netherlands) and 30 min later erythrocytes were *in vivo* labeled by intravenous injection of 740 MBq 99 mTc isotope. The heart chambers were imaged using a D-SPECT camera (Spectrum Dynamics, Israel) equipped with collimated, pixilated cadmium zinc telluride crystals detectors allowing rapid (7 min) data acquisition with superior spatial resolution. RV end-systolic and end-diastolic volumes were measured from three-dimensional reconstructed chambers using semiautomatic plug-in software (QBS Cedars-Sinai, Los Angeles, CA) by a single experienced physician.

### Statistical analysis

Data are presented as mean ± standard deviation, median with interquartile ranges (IQRs), or frequency (percent). Unpaired *t*-test or Mann–Whitney test were used to compare continuous variables between groups as appropriate. Cox univariate and multivariable models were used to test the effect of analyzed variables on prognosis. Event-free survival of patients was analyzed by Kaplan–Meier analysis with log-rank test comparison between groups. Calculations were performed using JMP 11 (SAS Institute Inc., Cary, NC) and R (Vienna, Austria). Regression package for R (version 2020.12.08) was used to compare the area under the curve (AUC) of the Cox Proportional Hazard Regression models at four different time points.

## Results

### Patients

A group of 836 patients with HFrEF were enrolled in the study. Over the follow up of 3.07 (IQRs 1.11; 4.89) years 508 patients (60.8%) experienced an adverse outcome (death, urgent heart transplantation, MCS implantation). Furthermore, 35 patients (4.2%) underwent HTx as non-urgent recipients. Patients achieved a high degree of guideline-directed pharmacotherapy and device therapy—78.5% had ACEi/ARB, 87.4% beta-blockers, 76.7% mineralocorticoid receptor antagonist, 57.0% ICD, Table 1.

**Table 1:** Patients characteristics.

	Whole cohort (*n* = 836)	RVGDs <20 (*n* = 204)	RVGDs 20–40 (*n* = 172)	RVGDs 40–60 (*n* = 183)	RVGDs >60 (*n* = 277)	*p* for trend
Age (years)	57.34 ± 11.28	58.19 ± 11.30	58.15 ± 10.85	57.60 ± 11.17	55.92 ± 11.57	**0.02**
Males (%)	82.8	76.0	82.0	85.3	86.6	**0.002**
HF etiology (% ischemic)	49.9	48.3	45.6	47.5	56.1	0.06
BMI (kg.m^−2^)	27.82 ± 5.09	29.45 ± 4.92	28.32 ± 5.20	27.60 ± 4.66	26.49 ± 5.11	**<0.0001**
NYHA (2–4, %)	32.1/60.5/7.5	48.5/49.0/2.5	39.0/56.4/4.7	25.1/65.6/9.3	20.6/67.5/11.1	**<0.0001**
Na (mmol.L^−1^)	138.53 ± 3.58	138.81 ± 3.02	139.65 ± 3.29	138.55 ± 3.84	137.57 ± 3.73	**<0.0001**
BNP (ng.L^−1^)	464 (207; 1076)	167 (89; 333.2)	329 (171; 686)	691 (343; 1213)	990 (567; 1720)	**<0.0001**
SBP (mmHg)	116.30 ± 19.10	124.39 ± 18.68	120.82 ± 19.93	112.4 ± 18.24	109.88 ± 16.38	**<0.0001**
Hemoglobin (g.L^−1^)	140.85 ± 18.19	139.63 ± 15.85	140.22 ± 17.49	141.10 ± 18.01	141.99 ± 20.40	0.14
DM (%)	377 (45.1%)	70 (34.3%)	63 (36.6%)	90 (49.2%)	154 (55.6%)	**<0.0001**
eGFR (ml.min^.−1^.1.73 m^−2^)	68.92 ± 22.50	71.55 ± 23.38	70.13 ± 22.96	68.25 ± 21.58	66.60 ± 22.24	**0.01**
Previous cardiac surgery (*n*, %)	197, 23.6	21, 10.3	46, 26.7	58, 31.2	72, 26.0	**<0.0001**
Cardiac morphology and function						
LVEDD (mm)	69.42 ± 9.10	66.69 ± 8.69	69.24 ± 9.58	70.24 ± 8.66	71.03 ± 9.01	**<0.0001**
LVEF (%)	23.58 ± 5.80	27.33 ± 5.25	25.03 ± 5.66	22.49 ± 4.47	20.68 ± 5.25	**<0.0001**
RVD_1_ (mm)	40.62 ± 7.94	34.72 ± 5.22	37.50 ± 5.97	40.62 ± 6.22	46.91 ± 7.20	**<0.0001**
RV dysfunction grade (0–3, %)	32.1/22.67/33.29/11.33	100/0/0/0	39.0/59.3/1.7/0	0/48.1/51.4/0.6	0/0/66.1/33.9	**<0.0001**
TAPSE/PASP ratio (mm/mmHg)	0.40 ± 0.21	0.64 ± 0.26	0.46 ± 0.17	0.36 ± 0.12	0.27 ± 0.11	**<0.0001**
Mitral regurgitation (1–3, %)	24.8/40.7/34.5	45.1/38.2/16.7	25.0/45.4/29.7	19.7/44.8/35.6	13.0/37.6/49.5	**<0.0001**
Tricuspid regurgitation (1–3, %)	44.5/39.1/16.4	81.8/16.8/1.5	53.8/40.4/5.9	37.9/50.0/12.1	15.6/47.3/37.1	**<0.0001**
IVC (mm)	19.55 ± 5.75	16.18 ± 3.63	17.87 ± 5.02	19.20 ± 5.00	23.24 ± 5.79	**<0.0001**
Therapy						
ACEi/ARB (%)	78.5	84.8	80.8	78.6	72.5	**0.0008**
BB (%)	87.4	87.8	89.5	89.0	86.2	0.54
MRA (%)	76.7	74.5	73.3	79.7	79.0	0.13
Furosemide daily dose (mg)	80 (40; 125)	40 (40; 80)	60 (40; 120)	80 (40; 125)	100 (60; 165)	**<0.0001**
ICD any (%)	57.0	60.4	60.3	57.1	59.3	0.72
CRT any (%)	30.8	70.8	71.4	62.7	67.4	0.24
Follow-up						
Death (%)	320 (38.3%)	54 (26.5%)	62 (36.1%)	83 (45.4%)	121 (43.7%)	–
Urg. HTx (%)	105 (12.6%)	6 (2.9%)	16 (9.3%)	28 (15.3%)	55 (19.9%)	–
Norm. HTx %)	35 (4.2%)	5 (2.5%)	11 (6.4%)	10 (5.5%)	9 (3.3%)	
MCSi (%)	83 (9.9%)	15 (7.4%)	13 (7.6%)	18 (9.8%)	37 (13.4%)	–
Alive with no event (%)	293 (35.0%)	124 (60.8%)	70 (40.7%)	44 (24.0%)	55 (19.9%)	–

ACEi, angiotensin-converting enzyme inhibitor; ARB, angiotensin receptor blocker; BB, beta-blocker; BMI, body mass index; CAD, coronary artery disease; CRT, cardiac resynchronization therapy; eGFR, estimated glomerular filtration rate; Hb1Ac, glycated hemoglobin; HTx, heart transplantation; ICD, implantable cardioverter-defibrillator; IVC, inferior vena cava; LVEDD, left ventricular diameter in diastole; LVEF, left ventricular ejection fraction; MCSi, mechanical circulatory support implantation; MiR, mitral regurgitation; MLHFQ, Minnesota living with heart failure questionnaire; MRA, mineralocorticoid receptor antagonist; NYHA, New York Heart Association; RV, right ventricular; RVD_1_, right ventricle basal diameter in apical four chamber view; TriR, tricuspid regurgitation; RVGDs, RV global dysfunction score.

Significant *p*-values are in bold.

### RV size and function

A total of 271 patients (32.4%) had preserved RV function, 190, 280 and 95 patients (22.7%, 33.5% and 11.4%) had mild, moderate and severe RV dysfunction, respectively.

RV dysfunction grade was associated with progressively deteriorating outcome (*p* < 0.001, Supplementary Figure S1). Compared to patients with preserved RV function, those with mild, moderate and severe RV dysfunction had 2-fold, 3-fold a 4.4-fold increased likelihood of an adverse outcome (HR 2.00 95% CIs 1.54–2.60 for mild RV dysfunction, HR 2.99 95% CI, 2.37–3.80 for moderate RV dysfunction and HR 4.42 95% CI, 3.29–5.91 for severe RV dysfunction, respectively, *p* < 0.0001). Similarly to RV dysfunction, RV size was also found to be significantly associated with adverse outcome (HR 1.02 95%CI, 1.01–1.04, *p* = 0.0002 after the adjustment for RV dysfunction grade). We have further analyzed whether an indexation of RV size brings any improvement in outcome prediction; absolute RV size was compared with RV size indexed to body surface area (RVD_1_i) that showed significantly higher area under the curve (AUC), Table 1.

Moreover, RVD_1_ was found to be independently associated with prognosis after the adjustment for TAPSE/PASP ratio (surrogate of RV-PA coupling), HR 1.02, 95% CI (1.006; 1.04), *p* = 0.009. RVD_1_ showed a loose but significant correlation with TAPSE/PASP ratio (*r*^2^ = 0.14, *p* < 0.0001); this correlation was tighter (*r*^2^ = 0.16, *p* < 0.0001) in patients without the history of pericardial opening (*n* = 632) and looser (*r*^2^ = 0.06, *p* = 0.02) in patients with the history of pericardial opening (*n* = 202).

### Intraobserver and interobserver variability testing

Intraoberver and interobserver variability was tested on the sample of 25 patients. Because of the restrospective nature of the study, saved loops were used for the analysis. Inraobserver variability for RVD_1_ was high (*r*^2^ = 0.97, average difference 0.92 mm, SD 0.86, Supplementary Figure S2A), RV function was categorized the same in 24 cases (in one case it RV function was assessed to have a moderate dysfunction in one case and mild dysfunction in the second assessment). Interobserver variability (assessed by independent experienced sonographer) was acceptable as well, *r*^2^ for RVD_1_ was 0.93 (average difference 1.52 mm, SD 0.82 mm, Supplementary Figure S2B), RV function was assessed in the same category in 22 cases (in all three cases where the disagreement was observed in the second evaluation RV function was assessed in the adjacent category—mild dysfunction vs. normal function, moderate dysfunction vs. mild dysfunction, and moderate dysfunction vs. severe dysfunction).

### Comparison of RV assessment by echocardiography and nuclear imaging

As echocardiography is a suboptimal method for evaluating RV size and function, we have performed a validation substudy; a subgroup of 89 patients (*n* = 36, 10, 29 and 14 with preserved RV function, mild, moderate and severe RV dysfunction, respectively) underwent RV size and function assessment by nuclear imaging. In all cases, echocardiography and scintigraphy was performed within 48 h during stable clinical conditions (stable p.o. medication). The scintigraphic examination was a part of a broader research project performer in our hospital, we have strived to have balanced number of patients in all subgroups of RV function. Patients with preserved RV function had a RV-ejection fraction (RVEF) of 53.64% (±3.93%), patients with mild, moderate and severe RV dysfunction had a RVEF of 46.10% (±3.63%), 34.93% (±4.01%), and 27.57% (±2.96%), respectively (Supplementary Figure S3A). Although RVEF assessment showed a mild overlap between groups, the discrimination by echocardiography seems to be satisfactory.

Further, we have evaluated the RVD_1_ measured by echocardiography and RV volume measured by nuclear imaging. Both parameters showed an acceptable degree of correlation—*r*^2^ = 0.76, *p* < 0.0001 (Supplementary Figure S3B). Thus, although imprecise, echocardiography seems to be an acceptable tool for RV size and function assessment in daily clinical practice.

### Combined parameter integrating both RV size and degree of dysfunction

**Figure 2:**
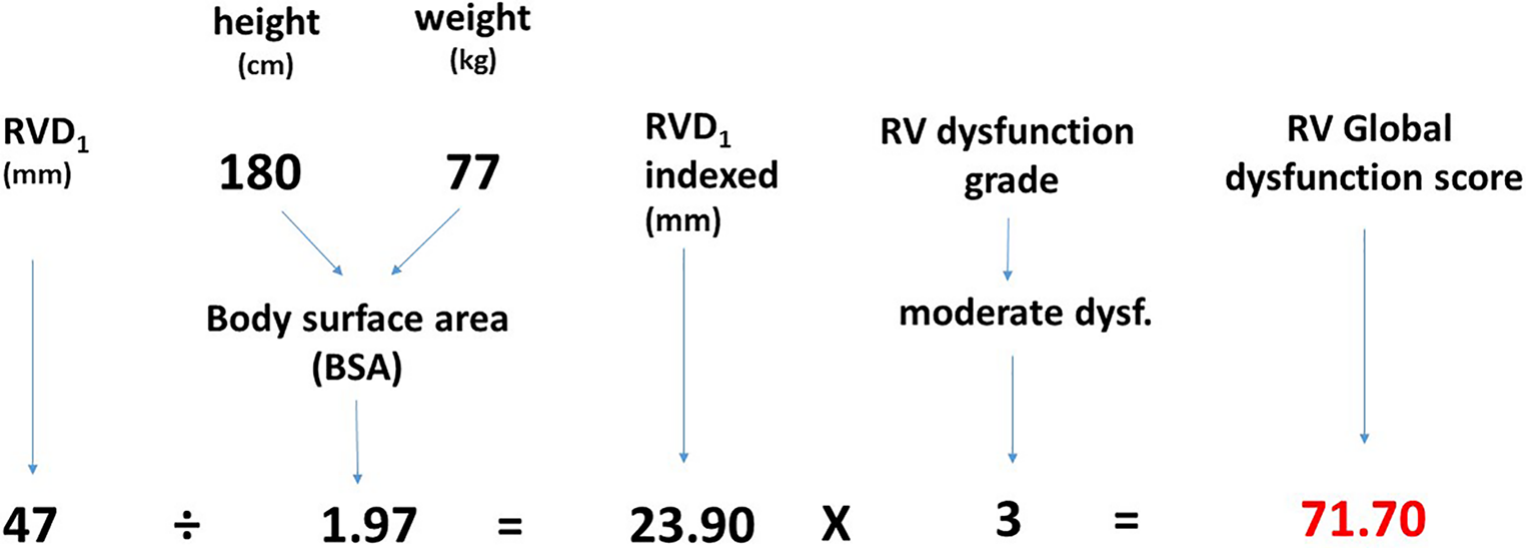
RV global dysfunction score. The calculation of RV global dysfunction score.

As RV size contributes to an adverse outcome independently of RV dysfunction, we suggest it should be considered as a manifestation of RV dysfunction. We have developed a parameter called “RV global dysfunction score” (RVGDs) that integrates the information about both RV size and the degree of dysfunction. It was calculated as a product of RVD_1_i and the degree of RV dysfunction (1 for preserved RV function, 2 for mild RV dysfunction, 3 for moderate RV dysfunction and 4 for severe RV dysfunction), Figure 2.

This closely reflects a progressive increase in hazard ratio with RV function worsening (HR of 2.00 for mild RV dysfunction compared to preserved RV function, 2.99 for moderate RV dysfunction and 4.42 for severe RV dysfunction, see the paragraph RV size and function). We have further compared the prognostic power of RVGDs with RV dysfunction grade only using AUC and found out that RVGDs was significantly superior (Table 2).

**Table 2:** Comparison of RV global dysfunction score with RV dysfunction grade only.

Time	AUC				
	RV global dysfunction	RV dysfunction grade	Delta AUC	95% CI	*p*
1st year	0.738	0.699	0.039	0.024; 0.055	**<0** **.** **0001**
2nd year	0.714	0.683	0.031	0.017; 0.044	**<0** **.** **0001**
3rd year	0.731	0.697	0.034	0.021; 0.047	**<0** **.** **0001**
4th year	0.737	0.703	0.034	0.021; 0.047	**<0** **.** **0001**

RV global dysfunction score was calculated as a product of RVD_1_i (indexed to BSA) multiplied by a factor of 1–4 (1 for preserved RV function, 2—mild RV dysfunction, 3- moderate RV dysfunction, 4- severe RV dysfunction). The area under the curve (AUC) of the Cox proportional hazard regression models was compared at four different time points.

Significant *p*-values are in bold.

### RV global dysfunction score

The contribution of RV dilatation and RV dysfunction grade on outcome was analyzed more in detail. We have compared patients with better RV function but more dilated RV with those with worse RV function but smaller RV size. Patients were divided into four groups according to RVGDs (<20, 20–40, 40–60 and >60); these intuitive cut-off values tightly reflect the distribution of RVGDs (median value of 43.9, IQRs 20.16 and 68.44). A total of 204, 172, 183 and 277 patients were involved in the respective subgroups (Table 1).

**Figure 3:**
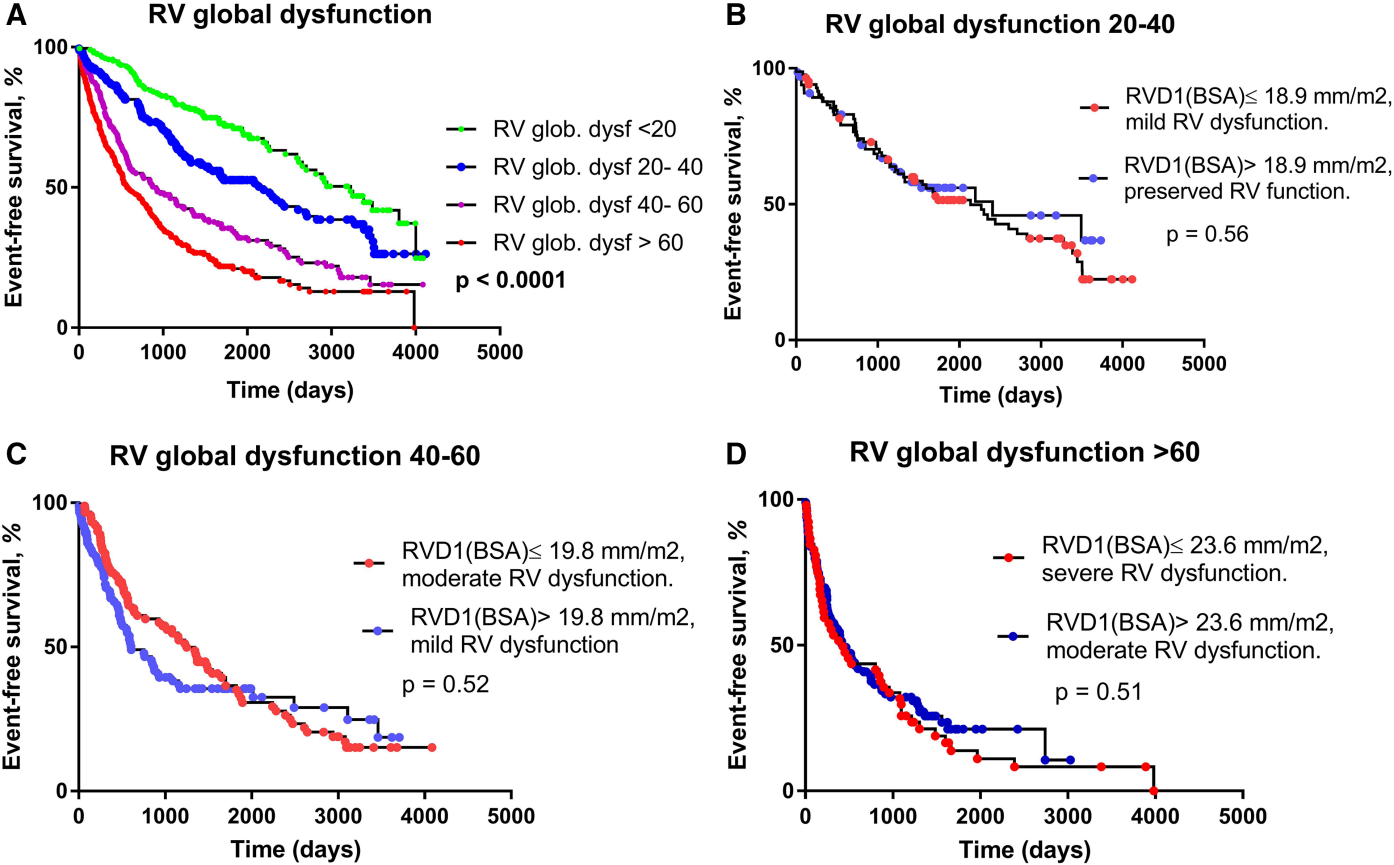
The relationship between RV global dysfunction score and prognosis. (**A**) Kaplan-Meier analysis of event-free survival according to RV global dysfunction score. (**B**) RV global dysfunction 20–40; RVD_1_i median = 18.9 mm/m^2^. RVD_1_i ≤ 18.9 mm/m^2^ and mild RV dysfunction (*N* = 84), RVD_1_i > 18.9 mm/m^2^ and preserved RV function (*N* = 67). (**C**) RV global dysfunction 40–60; RVD_1_i median = 19.8 mm/m^2^. RVD_1_i ≤ 19.8 mm/m^2^ and moderate RV dysfunction (*N* = 91), RVD_1_i > 19.8 mm/m^2^ and mild RV dysfunction (*N* = 88). D) RV global dysfunction >60; RVD_1_i median = 23.6 mm/m^2^. RVD_1_i ≤ 23.6 mm/m^2^ and severe RV dysfunction (*N* = 52), RVD RVD_1_i > 23.6 mm/m^2^ and moderate RV dysfunction (*N* = 99).

With increasing RVGDs, the outcome of patients progressively deteriorated (*p* < 0.0001, Figure 3A). In the first subgroup (RVGDs <20), all patients had preserved RV function. In the second subgroup (RVGDs 20–40), patients with RVD_1_i below median (≤18.9 mm/m^2^ for this subgroup) and mild RV dysfunction had the same outcome as those with RVD_1_i > 18.9 mm/m^2^, but preserved RV function (Figure 3B). Similarly, in the third subgroup (RVGDs 40–60), patients with RVD_1_i below median (≤19.8 mm/m^2^ for this subgroup) and moderate RV dysfunction had similar outcome as those with RVD_1_i > 19.8 mm/m^2^ but mild RV dysfunction (Figure 3C). Finally, in the fourth subgroup (RVGDs >60), patients with RVD_1_i below median (23.6 mm/m^2^ for this subgroup) and severe RV dysfunction had similar outcome as those with RVD_1_i > 23.6 mm/m^2^ but moderate RV dysfunction (Figure 3D). In order to exclude that the impact of RV size was in fact caused by more severe tricuspid regurgitation or larger degree of pulmonary hypertension, we have performed Cox multivariable regression that revealed that RV global dysfunction score was associated with adverse outcome even when adjusted for tricuspid regurgitation severity and the degree of pulmonary hypertension (Table 3).

**Table 3:** The impact of RV global dysfunction, pressure and volume overload on outcome.

	Univariable analysis			Multivariable analysis		
	HR	CI	*p*	HR	CI	*p*
RV global dysfunction, (100 units)	1.017	1.01–1.02	**<0.0001**	1.01	1.009–1.017	**<0.0001**
Tricuspid regurgitation severity, (1–3)	1.71	1.52–1.93	**<0.0001**	1.19	1.01–1.41	**0.04**
Estimated sPAP, (mmHg)	1.026	1.019–1.033	**<0.0001**	1.01	1.007–1.02	**0.0002**

Significant *p*-values are in bold.

With increasing RVGDs, patients were older, more often males, had more severe LV dysfunction (lower LV-ejection fraction) and enlarged LV cavity, more severe mitral and tricuspid regurgitation, lower plasma sodium and higher BNP level, worse renal function and were more often diabetic. Nevertheless, RV global dysfunction score was associated with an adverse outcome even after the adjustment for all these variables (Table 4).

**Table 4:** The impact of RV global dysfunction and other variables on outcome.

	Univariable analysis			Multivariable analysis		
	HR	CI	*p*	HR	CI	*p*
RV global dysfunction (100 units)	6.18	4.60–8.28	**<0.0001**	2.23	1.46–3.53	**0.0003**
Sex, (males vs. females)	1.87	1.44–2.47	**<0.0001**	1.51	1.13–2.05	**0.005**
LVEF, (%)	0.94	0.93–0.96	**<0.0001**	0.998	0.979–1.019	0.90
LVEDD, (mm)	1.02	1.02–1.04	**<0.0001**	1.01	1.0004–1.03	**0.04**
Mitral regurgitation, (1–3)	1.51	1.34–1.70	**<0.0001**	1.19	1.03–1.38	**0.02**
Tricuspid regurgitation, (1–3)	1.71	1.52–1.93	**<0.0001**	1.10	0.95–1.29	0.21
eGFR (ml.min^.−1^.1.73 m^−2^)	0.99	0.987–0.995	**<0.0001**	0.994	0.989–0.998	**0.005**
DM, (present vs. absent)	1.75	1.47–2.09	**<0.0001**	1.52	1.26–1.85	**<0.0001**
Na, (mmol/L)	0.91	0.89–0.93	**<0.0001**	0.96	0.94–0.98	**0.0005**
BNP, (100 ng/L)	1.07	1.06–1.08	**<0.0001**	1.05	1.03–1.06	**<0.0001**

Significant *p*-values are in bold.

## Discussion

This study shows that RV dilatation in HFrEF patients independently adds to an adverse outcome and should be considered as a marker of impaired RV function per se. Integrating the information about RV size and degree of dysfunction into one score parameter reflects more accurately the degree of RV disease.

The assessment of RV function in HFrEF patients is extremely important as RV dysfunction was repeatedly shown to be independently associated with impaired survival (2, 3). Currently, much effort is spent to improve the assessment of RV function, which is difficult due to its complex geometry. Tricuspid annular plane systolic excursion (TAPSE) and TDI-derived tricuspid lateral annular systolic velocity (sm-TDi) are long used parameters, but they both assess RV shortening in the longitudinal plane only (15) and are RV geometry-dependent. RV fractional area change (FAC) reflecting a difference between end-diastolic and end-systolic RV areas offers a 2-dimensional evaluation of RV function. In recent years, RV strain has been introduced and shown to better characterize the degree of RV dysfunction (16). Nevertheless, all these parameters focus solely on the property of RV myocardium without taking RV size into account. RV size as well has been shown to have prognostic value in HFrEF patients (8). Current guidelines, however, consider RV size and function as separate entities (11).

RV dysfunction and dilatation are thought to have different pathophysiological background, which is likely the reason why they are evaluated as separate entities. In the situation of pressure overload (pulmonary artery hypertension, PAH) RV initially responds with homeometric remodeling characterized with preserved volume, concentric hypertrophy and normal or only slowly declining RV function. When this adaptive remodeling is exhausted, progressive RV dilatation occurs (heterometric remodeling) (17). In HFrEF patients, the etiology of RV dilatation is likely much more diverse and in many cases can be a direct consequence of underlying pathology (coronary artery disease, dilated cardiomyopathy). RV dilatation thus does not seem to be a final stage of RV disease, it occurs rather independently of RV dysfunction. In our study, we have shown that RV size *per se* is a specific manifestation of RV dysfunction; patients with lower degree of RV dysfunction but larger RV size had similar outcome as those with worse RV dysfunction but smaller RV size. Importantly, this phenomenon is independent of tricuspid regurgitation severity (volume overload) and degree of pulmonary hypertension (pressure overload). The negative prognostic impact of larger RV size is thus attributable neither to more severe tricuspid regurgitation nor pulmonary hypertension. Currently, the estimates of RV systolic function are being replaced by surrogates reflection RV-PA coupling that can be noninvasively estimated as the TAPSE/PASP ratio. Increased RV size leads to increased wall stress that is an important determinant of oxygen consumption (18). Increased oxygen demand can result in periods of ischemia, possibly triggering ventricular arrhythmias. Alternatively, increased oxygen demand results in lower RV contraction efficiency that may ultimately lead to RV pump failure and pump failure death. In patients with HFrEF and secondary pulmonary hypertension, RV dilatation was a predictor of unfavorable right ventricle-to-pulmonary artery coupling (19), which was associated with markedly worse mortality.

In our study we have demonstrated that the combined parameter integrating the information about both RV size and the degree of dysfunction provides improved information about the prognosis compared with the degree of RV dysfunction alone. To the best of our knowledge, it is the first study testing this concept. Our data suggest that rather than focusing on RV dysfunction grade only, “RV disease” is more complex and RV size needs to be taken into account as well. As it is a retrospective echocardiographic analysis (although using prospectively enrolled patients), we used parameters available in all patients (RVD_1_, RV dysfunction grade assessed semi-quantitatively). Parameters assessing longitudinal RV function (TAPSE, Sm-TDi) are known to be reduced after cardiac surgery involving pericardial opening (20, 21). As a significant portion of patients (23.6%) have undergone previous cardiac surgery, using longitudinal parameters (TAPSE, Sm-TDi) to construct RV global dysfunction score seemed severely biased. Importantly, TAPSE/PASP ratio seems to be a better prognostic marker than crude RV dysfunction. Creating a combined score using RV size and TAPSE/PASP ratio (instead of RV dysfunction) might provide even better results than a score combining RV size and RV dysfunction. However, answering this question is not possible based on our data as TAPSE/PASP ratio was not available in a substantial portion of patients. It would most likely require a separate analysis of patients with/without the history of previous cardiac surgery. On the other hand, RVD_1_ and RV dysfunction grade (assessed semi-quantitatively) are parameters that can be established with a reasonable degree of precision even in patients with very poor acoustic windows. Importantly, the coefficients used for the construction of RVGDs very closely reflect the increasing risk of an adverse outcome. RV function can be associated with renal dysfunction through increased central venous pressure and decreased renal perfusion. Although we have observed a loose association between eGFR and RV function (assessed by both semiquantitatively as well as by RV global dysfunction score, *p* for trend 0.02 and 0.01, *r*^2^ = 0.007 and 0.008, respectively), RV function and eGFR were independently associated with impaired outcome. Therefore, RV function seems to be associated with prognosis regardless of renal function.

Naturally, a combination of different parameters reflecting RV function (FAC, RV strain) and size (RV end-diastolic or end-systolic volumes measured by 3D-echocardiography) might be superior to parameters chosen in the current study. Nevertheless, this is a proof-of-concept study showing that an integration of RV size and degree of dysfunction more accurately reflects the degree of RV disease. This new concept of RV evaluation should be further validated in other cohorts and it needs to be further investigated whether it is a general concept valid for other conditions associated with RV dysfunction (HFpEF, PAH).

## Limitations

Apical 4-chamber view was used for RVD_1_ measurement, RV global dysfunction score may lack generalizability if RVD_1_ was assessed using RV-dedicated 4-chamber projection.

Only a part of echocardiograms were stored electronically and thus available for off-line analysis. Fractional area change (FAC) was not routinely measured in all patients, so the RV global dysfunction score based on quantitative parameters could have not been obtained. Similarly, LV/RV strain parameters were measured only in a minority of patients. Patients were treated not only conservatively (i.e., by optimal pharmacotherapy and ICD/CRT device implantation), but some of them underwent heart transplantation or implantation of mechanical circulatory support, which may bias outcome analysis. As the patients were enrolled between 2009 and 2016, none were treated with sacubitril-valsartan or SGLT2 inhibitors at the time of enrollment. Sacubitril-valsartan was first reimbursed for HFrEF patients in 2018 and SGLT2i in 2021 and virtually no patients with HFrEF were treated with these agents before the reimbursement. Although patients were prospectively enrolled, not all of them were followed in our hospital, so the information about changes in pharmacotherapy throughout the follow-up period is not available from all the patients. Similarly, data about cardiac decompensation were not available from all the patients, so it was not possible to analyze this endpoint. Our study cohort included rather young patients with more advanced HF; consequently, the results might not be fully applicable to patients with milder HF or to older patients.

## Conclusion

RV dilatation should be considered to be a manifestation of RV dysfunction in HFrEF patients. A parameter integrating the information about both RV size and the degree of dysfunction provides superior prognostic assessment than the degree of RV dysfunction only.

## Data Availability

The raw data supporting the conclusions of this article will be made available by the authors, without undue reservation.
