# Supplementary figures and images for: Right ventricular global dysfunction score: a new concept of right ventricular function assessment in patients with heart failure with reduced ejection fraction (HFrEF)
Source: Front Cardiovasc Med. 2023 Aug 4;10:1194174. doi: 10.3389/fcvm.2023.1194174 (PMC10436518; doi:10.3389/fcvm.2023.1194174)

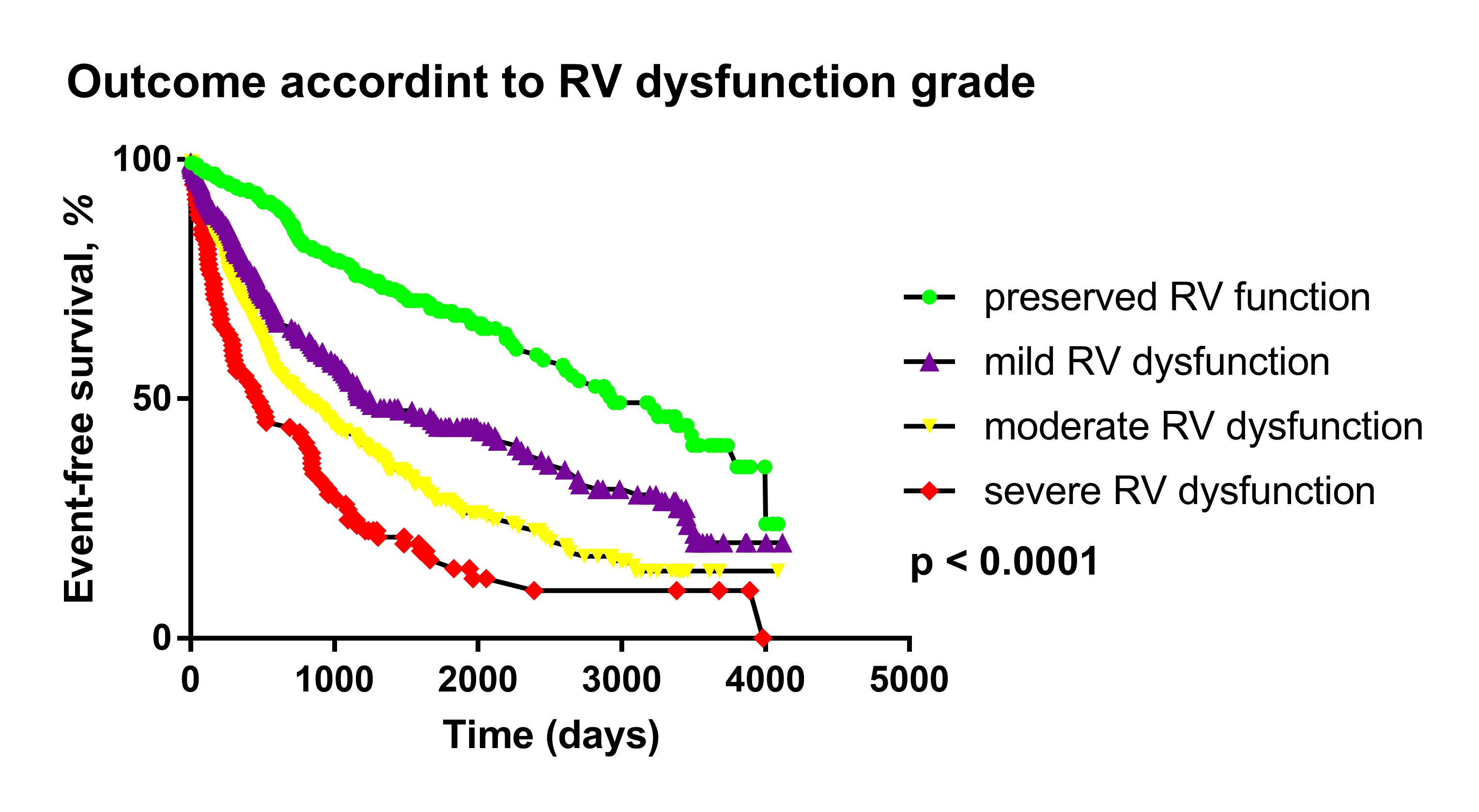

Supplement: Supplementary file 1 [file Image1.jpeg]

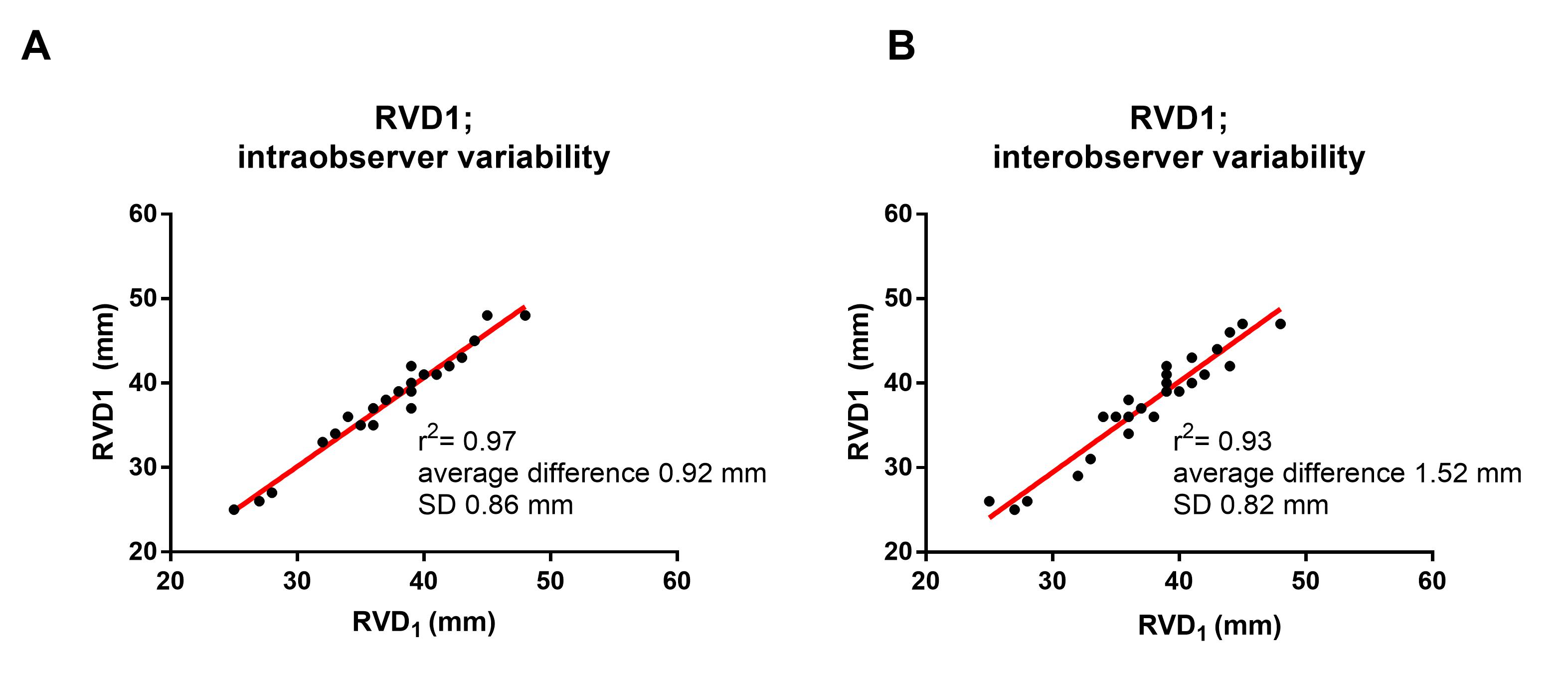

Supplement: Supplementary file 2 [file Image2.jpeg]

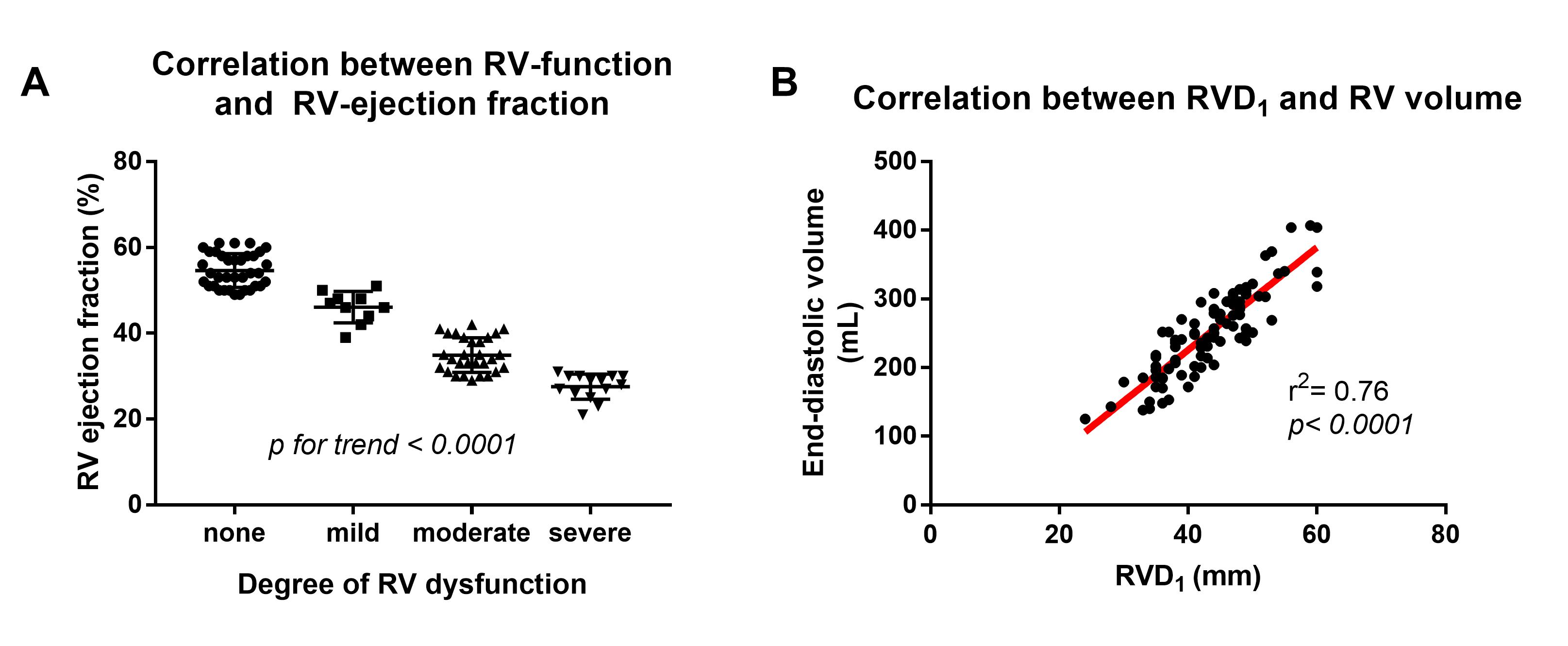

Supplement: Supplementary file 3 [file Image3.jpeg]
