# Supplementary material for: Right ventricular global dysfunction score: a new concept of right ventricular function assessment in patients with heart failure with reduced ejection fraction (HFrEF)
Source: Front Cardiovasc Med. 2023 Aug 4;10:1194174. doi: 10.3389/fcvm.2023.1194174 (PMC10436518; doi:10.3389/fcvm.2023.1194174)
Supplement: Supplementary file 4 [file Table1.docx]

**Online supplement, Table 1. Comparison of an absolute value of RVD_1_ with indexed to BSA**

| **Time** | **AUC** | | | | |
| --- | --- | --- | --- | --- | --- |
|  | **RVD_1_ (abs)** | **RVD_~~1~~_ (BSA)** | **Delta AUC** | **95% CI** | **P** |
| **1st year** | 0.677 | 0.709 | 0.032 | 0.011; 0.052 | **0.003** |
| **2nd year** | 0.657 | 0.682 | 0.025 | 0.008; 0.042 | **0.004** |
| **3rd year** | 0.676 | 0.703 | 0.027 | 0.011; 0.043 | **0.001** |
| **4th year** | 0.680 | 0.702 | 0.022 | 0.005; 0.039 | **0.012** |

The area under the curve (AUC) of the Cox proportional hazard regression models was compared at four different time points.
